# Thermoelectrocatalysis: an emerging strategy for converting waste heat into chemical energy

**DOI:** 10.1093/nsr/nwae036

**Published:** 2024-01-25

**Authors:** Yuqiao Zhang, Shun Li, Jianming Zhang, Li-Dong Zhao, Yuanhua Lin, Weishu Liu, Federico Rosei

**Affiliations:** Institute of Quantum and Sustainable Technology (IQST), School of Chemistry and Chemical Engineering, Jiangsu University, China; Institute of Quantum and Sustainable Technology (IQST), School of Chemistry and Chemical Engineering, Jiangsu University, China; Institute of Quantum and Sustainable Technology (IQST), School of Chemistry and Chemical Engineering, Jiangsu University, China; School of Materials Science and Engineering, Beihang University, China; State Key Laboratory of New Ceramics and Fine Processing, School of Materials Science and Engineering, Tsinghua University, China; Department of Materials Science and Engineering, Southern University of Science and Technology, China; Centre for Energy, Materials and Telecommunications, Institut national de la recherche scientifique, Canada

## Abstract

This perspective defines and explores an innovative waste heat harvesting strategy, thermoelectrocatalysis (TECatal), emphasizing materials design and potential applications in clean energy, environmental, and biomedical technologies.

Waste heat emissions are universally present in the environment and industrial production. Innovations aimed at efficiently utilizing low-grade thermal energy have long been desired. Among these, thermoelectric materials can directly convert heat into electricity based on the Seebeck effect. The overall performance of a thermoelectric material is assessed through a dimensionless figure of merit, *ZT*, represented as *ZT* = *S*^2^⋅*σ*/*κ*, where *S* is the Seebeck coefficient, *σ* is the electrical conductivity and *κ* is the thermal conductivity. In the pursuit of high conversion efficiency, recent years have witnessed the emergence of many promising thermoelectric materials with notable *ZT* values (>2).

In addition to research focused on thermal-electrical power generation, there has been a growing interest in exploring the direct coupling of the thermoelectric effect with catalytic processes. Pioneering studies from the 1970s demonstrated the integration of thermoelectric devices with electrochemical cells for water splitting [1]. More recent investigations have unveiled intriguing intrinsic temperature-gradient-induced catalytic reactions in nanostructured thermoelectric materials [2,3]. Based on these findings, we introduce the concept of thermoelectrocatalysis (TECatal), which combines the thermoelectric effect with surface catalysis processes. Various types of heat, even those with relatively small temperature differences from the natural environment, industrial production and daily life, can be converted into chemical energy through TECatal materials to drive diverse catalytic reactions in areas of clean energy (e.g. water splitting, CO_2_ reduction and batteries), environmental remediation (e.g. degradation of water pollutants and air purification), biomedical technologies (e.g. antimicrobial systems and cancer therapy) and materials synthesis (e.g. value-added organics) [1,3–6].

As an emerging type of catalyst, we briefly summarize and propose several potential working modes while elucidating the underlying principles of different TECatal systems: (**i) Hybrid structure mode**. The temperature-gradient (Δ*T*)-induced built-in electric field in thermoelectric materials can modulate the work function or band alignment of surface-loaded noble metal or semiconductors (Fig. 1a) [4]; (**ii) Single-phase mode**. Thermoelectric nanostructures directly act as catalysts to induce chemical reactions driven by Δ*T* (Fig. 1b) [2,3]. This leads to a chemical potential difference inside the thermoelectric material, creating a built-in electric field that effectively drives the migration of electrons and holes toward opposite directions to trigger surface redox reactions; (**iii) P-N heterojunction mode.** We propose a novel TECatal mode using thermoelectric P-N nanojunctions (Fig. 1c, left), where thermally excited charge carriers are separated by the built-in electric field near the junction under a homogeneous heating environment, similar to well-studied photovoltaic and photocatalytic mechanisms. The P-N heterostructure enhances charge separation through the directional migration of electrons and holes. Additionally, creating a temperature gradient along the junction can drive excited electrons (n-type) and holes (p-type) across the material towards opposite ends (Fig. 1c, right). Further theoretical and experimental studies are essential to validate this mode; (**iv) Thermogalvanic cell mode**. Recently, a thermoelectric-gel-based ionic gelatin matrix was proposed to realize a continuous concentration gradient of redox ions at the hot and cold sides, achieving a solar-to-hydrogen water-splitting efficiency of up to 0.4%, accompanied by a simultaneous thermopower of 8.2 mV K^−1^ [7]. In the future, the application of double chemically crosslinked networks to ionic thermogalvanic cells may enhance both mechanical toughness and power density [8], thereby advancing the application of thermogalvanic cells in TECatal. The detailed discussion about the recent progress of different TECatal modes is summarized in the supplementary data.

**Figure 1.**
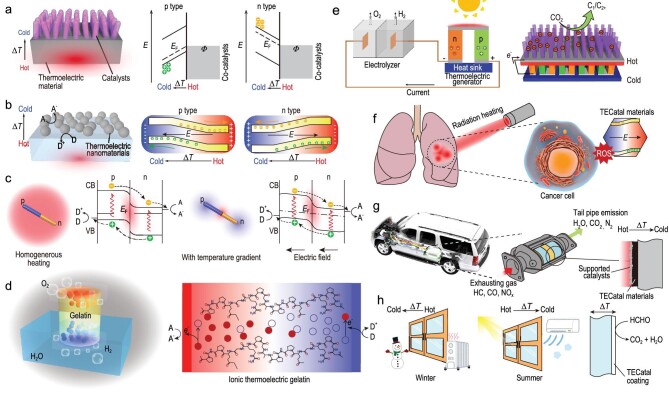
Working modes of TECatal systems: (a) hybrid structure mode (right, redrawn from [4]); (b) single-phase mode (right, redrawn from [3]); (c) p-n nanojunction mode and (d) thermogalvanic cell mode (redrawn from [7]). Potential applications of TECatal materials: (e) H_2_ production and CO_2_ reduction (redrawn from [1]); (f) tumor therapy; (g) vehicle tail gas treatment; (h) window glass coating for indoor air purification.

The overall thermal-to-chemical energy conversion process in TECatal involves two sequential steps: thermal to electrical energy (*η*_TE_) and electrical to chemical energy (*η*_EC_) conversion. Here, we tentatively propose several selection principles and design strategies for TECatal materials to achieve a high conversion efficiency of thermal-to-chemical energy: (**i) High *ZT* principle.** Certainly, a high Seebeck coefficient and low thermal conductivity are essential factors. Strategies such as element doping, nanojunction and nanostructure/quantum size effects can be employed to optimize TECatal efficiency by effectively modulating electron and phonon transport properties; (**ii) Band alignment principle.** Selecting and modulating TECatal materials with appropriate band gaps and band positions is crucial. For example, creating composite alloys with large band gap materials can widen the band gap of semimetal thermoelectric materials, thus matching the required chemical redox potential for driving desired catalytic reactions; (**iii) Microstructure/surface modulation principle.** Well-designed nanostructures with a large specific surface area, highly reactive exposed facets and appropriate surface modifications (e.g. loading co-catalysts or surface functional groups) are highly desirable; (**iv) Stability principle.** For practical applications, careful selection of material candidates with high chemical and thermal stability is necessary.

Future research efforts on TECatal materials are anticipated to stimulate a broader range of catalytic applications. This perspective tentatively proposes several application scenarios. First, by integrating thermoelectric modules with photoelectrochemical cells, waste heat can be harnessed to drive water splitting and CO_2_ reductions (Fig. 1e) [1,5,9]. Second, recent efforts have focused on biomedical applications using thermoelectric nanomaterials [10]. Temperature fluctuations in organisms or electromagnetic irradiation can induce a temperature gradient, enabling TECatal reactions for cancer therapy (Fig. 1f). Third, the exhaust gas in automobile systems could be converted into non-toxic byproducts by utilizing the temperature difference inside and outside the exhaust pipe with TECatal materials, potentially replacing noble metal catalysts (Fig. 1g). Finally, thin film or nanostructured TECatal materials could act as coatings on the inner side of window glass, serving as smart indoor air purification and antimicrobial components (Fig. 1h).

To date, thermoelectric materials have been extensively studied for thermocouples, electricity generators and refrigerators. The emerging concept of ‘TECatal’ introduces numerous unforeseen opportunities. Despite recent progress, the understanding of the fundamental mechanisms of TECatal is still in its early stages. The promising prospects of TECatal in various catalytic application fields, such as energy, environmental and biomedical technologies driven by relatively small temperature differences, define an opportunity to merge waste heat harvesting with green catalysis processes.

## Supplementary Material

nwae036_Supplemental_File
