# Supplementary material for: Thermoelectrocatalysis: an emerging strategy for converting waste heat into chemical energy
Source: Natl Sci Rev. 2024 Jan 25;11(4):nwae036. doi: 10.1093/nsr/nwae036 (PMC10911810; doi:10.1093/nsr/nwae036)
Supplement: nwae036_Supplemental_File [file nwae036_supplemental_file.pdf]

Supporting information

Thermoelectrocatalysis: An Emerging Strategy for Converting Waste Heat into Chemical Energy

Yuqiao Zhang,[1] Shun Li,[1,*] Jianming Zhang,[1] Lidong Zhao,[2] Yuanhua Lin,[3,*] Weishu Liu,[4,*] and Federico Rosei [5,*]

[1] *Institute of Quantum and Sustainable Technology (IQST), School of Chemistry and Chemical Engineering, Jiangsu University, Zhenjiang 212013, Jiangsu, China*

[2] *School of Materials Science and Engineering, Beihang University, Beijing 100191, China*

[3] *State Key Laboratory of New Ceramics and Fine Processing, School of Materials Science and Engineering, Tsinghua University, Beijing, 100084, China*

[4] *Department of Materials Science and Engineering, Southern University of Science and Technology, Shenzhen, Guangdong 518055, China*

[5] *Centre for Energy, Materials and Telecommunications, Institut national de la recherche scientifique, 1650 Boul. Lionel-Boulet, Varennes, QC, J3X 1S2, Canada*

∗ **Corresponding authors.**
E-mails: shun@ujs.edu.cn; liuws@sustech.edu.cn; linyh@tsinghua.edu.cn; federico.rosei@inrs.ca

Herein we present details of reported working modes of TECatal systems and highlight the significant advances achieved in each mode.

1. Thermoelectric device and electrochemical cell mode

This mode can be traced back to 1970th. In this mode, external thermoelectric generators were integrated with electrochemical cell, performing as a “battery” to initiate photochemical reactions [1, 2]. Using solar energy as the heating source, the total hydrogen production could be increased by 20%. In 2013, Yang *et al.* developed a hybrid energy cell which combines a triboelectric nanogenerator, a thermoelectric cell, and a solar cell for water splitting. This innovative design enables the simultaneous harvesting of mechanical, thermal, and solar energies [3]. Chen *et al.*

coupled thermoelectric microconverter with microbial electrolysis cell, obtaining a relatively high cathodic hydrogen recovery (81–95%) and hydrogen yield (2.7 mol/mol acetate) [4]. In addition to the separated system, Yuan *et al.* integrated bifunctional Ni@NCNTs/NF-L, which performs as both photothermal conversion layer and efficient hydrogen evolution reaction (HER) electrocatalyst, with a thermoelectric generator, realizing the highly dense hybrid external thermoelectric-assisted electrocatalytic system [5]. Very recently, Pornrungroj *et al.* demonstrated the directly utilization of waste heat to benefit photoelectrochemical (PEC) devices [6]. The additional thermoelectric bias voltage enhanced the product output and photocurrent density. This novel approach enables unassisted overall water splitting, even for PEC systems with insufficient photovoltage.

2. Intrinsic TECatal mode

(1) Hybrid structure. In 2018, Achour *et al*. introduced the concept of thermoelectric promotion of catalysis (TEPOC), based on non-Faradaic electrochemical modification of catalytic activity (NEMCA) [7, 8]. It enables the controlled *in-situ* introduction of promoters on catalyst surfaces under operating conditions. NEMCA, involving a reversible change in the catalytic properties of metal catalysts deposited on solid electrolytes, can be achieved by applying a small external electric current or voltage. Thermoelectric materials serve as an excellent platform for altering the effective work function of catalyst particles, leading to a significant increase in catalytic activity. This concept was applied to ethylene oxidation and CO_2 hydrogenation, utilizing BiCuSeO as a support and promoter. In CO_2 hydrogenation, an notable CO_2 conversion rate of 48.4% to CO with 100% selectivity, was achieved. Additionally, Xu *et al*. developed a multi-field driven hybrid catalyst Pt/ZnO nanorod arrays/$Bi_{1-x}Er_xCuSeO$ for CO_2 reduction, which can take advantage of both photocatalysis and TECatal. Utilizing a synergetic photothermoelectric effect, the maximum CO production rate of 2.91 μmol g^{-1} h^{-1} at 423 K was achieved [9].

(2) Single-phase. This intrinsic TECatal working mode was firstly reported by Sharifi, *et al*. in 2017. Nanostructured thermoelectric materials directly acted as catalyst, promoting electrochemical redox through internally generated electron-hole pairs [10].

The built-in electric field induced by a temperature gradient was proposed to enhance the separation of electron-hole pairs, simultaneously modifying the redox potential on the material's surface, which improved the HER and antibacterial processes [10, 11].

(3) Thermogalvanic cell. The recently proposed ionic gelatin thermogalvanic mode is inspired by the ionic thermoelectric materials [12, 13]. Wang *et al*. introduced an in-situ photocatalytically enhanced redox reaction that generates hydrogen and oxygen, establishing a continuous concentration gradient of redox ions in thermogalvanic devices [14]. The system exhibited a thermopower of 8.2 millivolts per kelvin and a solar-to-hydrogen efficiency of up to 0.4%. They further developed a generator with a large area of 112 cm^2, which yielded an open-circuit voltage of 4.4 V and a power of 20.1 mW. After 6 hours' operation under natural light, 0.5 mol of hydrogen and 0.2 mol of oxygen were produced.

Reference

[1] T. Ohta, S. Asakura, M. Yamaguchi, N. Kamiya, N. Gotgh, T. Otagawa, Photochemical and thermoelectric utilization of solar energy in a hybrid water-splitting system, Int. J. Hydrogen Energy, 1 (1976) 113-116.

[2] T. Ohta, N. Kamiya, M. Yamaguchi, N. Gotoh, T. Otagawa, S. Asakura, System efficiency of a water-splitting system synthesized by photochemical and thermoelectric conversion of solar energy, Int. J. Hydrogen Energy, 3 (1978) 203-208.

[3] Y. Yang, H. Zhang, Z.-H. Lin, Y. Liu, J. Chen, Z. Lin, Y.S. Zhou, C.P. Wong, Z.L. Wang, A hybrid energy cell for self-powered water splitting, Energy Environ. Sci., 6 (2013) 2429-2434.

[4] Y. Chen, M. Chen, N. Shen, R.J. Zeng, H2 production by the thermoelectric microconverter coupled with microbial electrolysis cell, Int. J. Hydrogen Energy, 41 (2016) 22760-22768.

[5] H. Yuan, F. Liu, G. Xue, H. Liu, Y. Wang, Y. Zhao, X. Liu, X. Zhang, L. Zhao, Z. Liu, H. Liu, W. Zhou, Laser patterned and bifunctional Ni@N-doped carbon nanotubes as electrocatalyst and photothermal conversion layer for water splitting driven by thermoelectric device, Appl. Catal., B, 283 (2021).

[6] C. Pornrungroj, V. Andrei, E. Reisner, Thermoelectric–Photoelectrochemical Water Splitting under Concentrated Solar Irradiation, J. Am. Chem. Soc., 145 (2023) 13709-13714.
[7] A. Achour, K. Chen, M.J. Reece, Z. Huang, Tuning of Catalytic Activity by Thermoelectric Materials for Carbon Dioxide Hydrogenation, Adv. Energy Mater., 8 (2018) 1701430.
[8] A. Achour, J. Liu, P. Peng, C. Shaw, Z. Huang, In Situ Tuning of Catalytic Activity by Thermoelectric Effect for Ethylene Oxidation, ACS Catal., 8 (2018) 10164-10172.
[9] Y. Xu, J. Han, Y. Luo, Y. Liu, J. Ding, Z. Zhou, C. Liu, M. Zou, J. Lan, C.w. Nan, Y. Lin, Enhanced CO_2 Reduction Performance of BiCuSeO‐Based Hybrid Catalysts by Synergetic Photo‐Thermoelectric Effect, Adv. Funct. Mater., 31 (2021) 2105001.
[10] T. Sharifi, X. Zhang, G. Costin, S. Yazdi, C.F. Woellner, Y. Liu, C.S. Tiwary, P. Ajayan, Thermoelectricity Enhanced Electrocatalysis, Nano Lett., 17 (2017) 7908-7913.
[11] Y.-J. Lin, I. Khan, S. Saha, C.-C. Wu, S.R. Barman, F.-C. Kao, Z.-H. Lin, Thermocatalytic hydrogen peroxide generation and environmental disinfection by Bi_2Te_3 nanoplates, Nat. Commun., 12 (2021) 1-11.
[12] C.-G. Han, X. Qian, Q. Li, B. Deng, Y. Zhu, Z. Han, W. Zhang, W. Wang, S.-P. Feng, G. Chen, W. Liu, Giant thermopower of ionic gelatin near room temperature, Science, 368 (2020) 1091-1098.
[13] Y. Li, Q. Li, X. Zhang, B. Deng, C. Han, W. Liu, 3D Hierarchical Electrodes Boosting Ultrahigh Power Output for Gelatin‐KCl‐FeCN4−/3− Ionic Thermoelectric Cells, Adv. Energy Mater., 12 (2022).
[14] Y. Wang, Y. Zhang, X. Xin, J. Yang, M. Wang, R. Wang, P. Guo, W. Huang, A.J. Sobrido, B. Wei, X. Li, In situ photocatalytically enhanced thermogalvanic cells for electricity and hydrogen production, Science, 381 (2023) 291-296.
